# OSTEOSARCOPENIC OBESITY AND VITAMIN D IN U.S. ADULTS AGES 50 AND OLDER: A CROSS-SECTIONAL STUDY

**DOI:** 10.1093/geroni/igz038.1789

**Published:** 2019-11-08

**Authors:** Barbara S Saltzman, Kevin C Kenney

**Affiliations:** University of Toledo, Toledo, Ohio, United States

## Abstract

Osteosarcopenic Obesity (OSO) is the concurrent presence of obesity (excess body fat), sarcopenia (low muscle mass) and osteoporosis (low bone mineral density). Low levels of serum vitamin D (vitD) have been associated with each disease and their simultaneous presence. We examined this association in adults aged ≥50 years from the 2001-2006 survey cycles of the National Health and Nutrition Examination Survey (NHANES) (N=3791). SAS v9.4 complex survey procedures were used. Demographics and vitD status were compared across OSO components (0,1,2,3) by chi-squared tests and ANOVAs. Odds ratios and 95% confidence intervals for the association between vitD status and increasing number of OSO components were calculated using multinomial logistic regression (alpha=0.05). The mean age was 65.0 years (30% were older adults ≥ 65), 45.6% were female, and prevalences for obesity, osteoporosis, sarcopenia and OSO were 84.7%, 8.0%, 16.3%, and 1.8%, respectively. Additionally, 30.9% of the sample was vitD deficient. After adjusting for supplement use, and race, women with 1 or 2, and men with 1,2 or 3 OSO components were more likely to be vitD deficient than sufficient, compared to those with none [women: 2.8(1.4-5.7), 2.1(1.4-6.8), and men: 2.3 (1.5-3.6), 3.5(2.1-5.9) and 6.3(1.0-38.5), respectively]. Vitamin D status was associated with having one or two OSO components in women, and OSO and components in men, after adjusting for supplement use, sex, and race. These results suggest it is important to consider obesity, osteoporosis, and sarcopenia jointly with respect to vitamin D status in the chronic disease management of aging and older adults.

